# Altered gray‐to‐white matter tissue contrast in preterm‐born adults

**DOI:** 10.1111/cns.14320

**Published:** 2023-06-27

**Authors:** Benita Schmitz‐Koep, Aurore Menegaux, Juliana Zimmermann, Melissa Thalhammer, Antonia Neubauer, Jil Wendt, David Schinz, Marcel Daamen, Henning Boecker, Claus Zimmer, Josef Priller, Dieter Wolke, Peter Bartmann, Christian Sorg, Dennis M. Hedderich

**Affiliations:** ^1^ Department of Diagnostic and Interventional Neuroradiology Technical University of Munich; School of Medicine Munich Germany; ^2^ Technical University of Munich, School of Medicine, TUM‐NIC Neuroimaging Center Munich Germany; ^3^ Department of Diagnostic and Interventional Radiology University Hospital Bonn, Clinical Functional Imaging Group Bonn Germany; ^4^ Department of Neonatology and Pediatric Intensive Care University Hospital Bonn Bonn Germany; ^5^ Department of Psychiatry Technical University of Munich, School of Medicine Munich Germany; ^6^ Department of Psychology University of Warwick Coventry UK; ^7^ Warwick Medical School University of Warwick Coventry UK

**Keywords:** brain development, cerebral cortex, preterm birth, structural magnetic resonance imaging, tissue contrast

## Abstract

**Aims:**

To investigate cortical organization in brain magnetic resonance imaging (MRI) of preterm‐born adults using percent contrast of gray‐to‐white matter signal intensities (GWPC), which is an in vivo proxy measure for cortical microstructure.

**Methods:**

Using structural MRI, we analyzed GWPC at different percentile fractions across the cortex (0%, 10%, 20%, 30%, 40%, 50%, and 60%) in a large and prospectively collected cohort of 86 very preterm‐born (<32 weeks of gestation and/or birth weight <1500 g, VP/VLBW) adults and 103 full‐term controls at 26 years of age. Cognitive performance was assessed by full‐scale intelligence quotient (IQ) using the Wechsler Adult Intelligence Scale.

**Results:**

GWPC was significantly decreased in VP/VLBW adults in frontal, parietal, and temporal associative cortices, predominantly in the right hemisphere. Differences were pronounced at 20%, 30%, and 40%, hence, in middle cortical layers. GWPC was significantly increased in right paracentral lobule in VP/VLBW adults. GWPC in frontal and temporal cortices was positively correlated with birth weight, and negatively with duration of ventilation (*p* < 0.05). Furthermore, GWPC in right paracentral lobule was negatively correlated with IQ (*p* < 0.05).

**Conclusions:**

Widespread aberrant gray‐to‐white matter contrast suggests lastingly altered cortical microstructure after preterm birth, mainly in middle cortical layers, with differential effects on associative and primary cortices.

## Graphical abstract

Using structural magnetic resonance imaging, this study shows widespread lower gray‐to‐white matter tissue contrast in associative cortices, mainly in middle cortical layers, and higher contrast in primary cortex. Results suggest lastingly altered cortical microstructure after preterm birth with differential effects on associative and primary cortices.

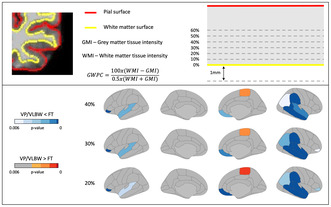

## 1 INTRODUCTION

Preterm birth (<37 weeks of gestation) is associated with altered brain development, such as lower gray and white matter volumes, aberrant cortical architecture, and disturbed white matter integrity, as measured using magnetic resonance imaging (MRI).^1^, ^2^, ^3^, ^4^, ^5^, ^6^, ^7^ On a microscopic level, various brain insults associated with preterm birth, for example hypoxia‐ischemia and inflammation, lead to disrupted development of specific cell populations such as pre‐oligodendrocytes (pre‐OLs) and subplate neurons (SPNs).^8^, ^9^, ^10^ SPNs are a largely transient neuronal population that play an essential role in cortical development.^11^, ^12^ They reach peak population size around 17–37 weeks GA, thus overlapping with preterm birth, and then decline in number.^11^, ^12^ SPNs are particularly vulnerable to hypoxia‐ischemia in the context of preterm birth, leading to SPN dysmaturation and disrupted cortical development.^8^, ^13^

Recently, the percent contrast of gray‐to‐white matter signal intensities (GWPC), sampled across different cortical layers, has been proposed as an in vivo proxy measure for irregular microstructural organization of the cortex, based on data from individuals with autism spectrum disorder.^14^ Lower gray‐to‐white matter contrast has also been reported in healthy aging, in patients with Alzheimer's disease and in patients with schizophrenia.^15^, ^16^, ^17^ Hence, this measure seems to serve as a biomarker of aging and age‐associated disease, and of neurodevelopmental and psychiatric disorders associated with abnormal SPN development.^14^, ^15^, ^16^, ^17^, ^18^, ^19^ Preterm birth has been associated with both an increased risk for accelerated brain aging and with SPN injury.^8^, ^13^, ^20^ Moreover, cortical architecture in preterm‐born individuals exhibits macrostructural alterations that last into adulthood, such as aberrant cortical folding or regionally lower cortical thickness (CTh).^21^, ^22^, ^23^, ^24^ However, the microstructural organization of the cortex in preterm‐born adults remains less clear. Therefore, we analyzed GWPC as an in vivo proxy measure for cortical organization in 86 adults born very preterm and/or with very low birth weight (VP/VLBW, <32 weeks of gestation and/or birth weight [BW] <1500 g) and 103 full‐term (FT) controls at 26 years of age. In line with findings in aging and age‐associated disease as well as other neurodevelopmental and psychiatric disorders associated with abnormal SPN development, such as autism spectrum disorder and schizophrenia, we hypothesized lower GWPC after preterm birth. Furthermore, it is known that IQ deficits after preterm birth persist into adulthood which is mediated by altered cortical architecture.^21^, ^22^, ^23^, ^24^, ^25^, ^26^, ^27^, ^28^ Therefore, we hypothesized that altered GWPC might be associated with lower full‐scale IQ in preterm‐born adults.

## 2 METHODS

### 2.1 Participants

Our study sample, the Bavarian Longitudinal Study, has been previously described^27^, ^28^, ^29^, ^30^, ^31^, ^32^, ^33^: In brief, 101 subjects born VP (<32 weeks of gestation) and/or with very low birth weight (VLBW, birth weight <1500 g; VP/VLBW) and 111 FT controls, which were randomly selected as control subjects within the stratification variables of sex and family socioeconomic status (SES), have been studied prospectively since birth and underwent MRI at 26 years of age (see Supplement S1 for more details). The MRI examinations took place at two sites: The Department of Neuroradiology, Klinikum rechts der Isar, Technische Universität München, (*n* = 145) and the Department of Radiology, University Hospital of Bonn (*n* = 67). The study was carried out in accordance with the Declaration of Helsinki and was approved by the local ethics committee of the Klinikum rechts der Isar, Technische Universität München and the University Hospital Bonn. All study participants gave written informed consent. They received travel expenses and a small payment for participation.

### 2.2 Birth variables

Gestational age (GA) was estimated from maternal reports on the first day of the last menstrual period and serial ultrasounds during pregnancy. In cases in which the two measures differed by more than 2 weeks, clinical assessment at birth with the Dubowitz method was applied.^34^ BW was obtained from obstetric records.^30^, ^35^ The duration of mechanical ventilation in days was computed from daily records by research nurses. Family SES was assessed through structured parental interviews within 10 days of childbirth. SES was computed as a weighted composite score based on the profession of the self‐identified head of each family together with the highest educational qualification held by either parent,^36^ resulting in three categories.

### 2.3 Cognitive performance in adulthood

To assess global cognitive performance at the age of 26, prior to and independent of the MRI examination, study participants were asked to complete a short version of the “Wechsler Intelligenztest für Erwachsene” (WIE), the German adaptation of the Wechsler Adult Intelligence Scale, third edition (WAIS‐III).^37^ This test was carried out by trained psychologists who were blinded to group membership and used to derive full‐scale IQ estimates.^27^, ^32^

### 2.4 MRI data acquisition

MRI data acquisition has been described previously^4^, ^23^: At both sites, Bonn and Munich, MRI data acquisition was performed on Philips Achieva 3 T TX systems or Philips Ingenia 3 T system using an 8‐channel SENSE head coil. Subject distribution among scanners was as follows: Bonn Achieva 3 T: 5 VP/VLBW, 12 FT, Bonn Ingenia 3 T: 33 VP/VLBW, 17 FT, Munich Achieva 3 T: 60 VP/VLBW, 65 FT, Munich Ingenia 3 T: 3 VP/VLBW, 17 FT. Across all scanners, sequence parameters were kept identical. Scanners were checked regularly to provide optimal scanning conditions and MRI physicists at the University Hospital Bonn and Klinikum rechts der Isar regularly scanned imaging phantoms to ensure within‐scanner signal stability over time. Signal‐to‐noise ratio was not significantly different between scanners (one‐way ANOVA with factor ‘scanner‐ID’ [Bonn 1, Bonn 2, Munich 1, Munich 2]; *F*(3,182) = 1.84, *p* = 0.11). A high‐resolution T1w 3D‐MPRAGE sequence (TI = 1300 ms, TR = 7.7 ms, TE = 3.9 ms, flip angle = 15°, field of view = 256 mm × 256 mm, reconstruction matrix = 256 × 256, reconstructed isotropic voxel size = 1 mm^3^) was acquired. All images were visually inspected for artifacts.

### 2.5 MRI processing and calculation of GWPC

Images saved as DICOMs were converted to Nifti‐format using dcm2nii.^38^ FreeSurfer v7.1.1 (http://surfer.nmr.mgh.harvard.edu/) was used to process the T1w images. These automated methods have been described in detail elsewhere and have been validated against histological data.^39^, ^40^, ^41^, ^42^, ^43^, ^44^ Following the approach by Andrews et al.,^14^ gray matter tissue intensities (GMI) were sampled at different percentile fractions of the total orthogonal distance projected from the white matter surface to the pial surface (0%, 10%, 20%, 30%, 40%, 50%, and 60%). White matter signal intensity (WMI) was sampled at 1.0 mm into the white matter from the white matter surface. Then, the formula provided by FreeSurfer was used to calculate tissue contrast as the percentage of GMI at projection fraction (j) to WMI at each cerebral vertex (i):

$${\text{GWPC}}_{\mathrm{ij}}=100\times \left({\mathrm{WMI}}_{i,1.\text{0mm}}–{\mathrm{GMI}}_{i,j}\right)/0.5\times \left({\mathrm{WMI}}_{i,1.\text{0mm}}+{\mathrm{GMI}}_{i,j}\right).$$

Hence, lower GWPC indicates lower contrast between GMI and WMI. This formula differs from other previously reported measures of tissue contrast that have used a ratio calculation (GMI/WMI) with higher values indicating lower contrast.^15^ The surface was then subdivided into 68 gyral‐based regions of interest (ROIs), 34 per hemisphere, using the Desikan–Killiany Atlas,^45^ and GWPC was extracted within these ROIs. Furthermore, CTh values were extracted within these ROIs. The FreeSurfer “recon‐all” pipeline failed in three cases due to limited image quality. Output quality was assessed using FreeSurfer's QA tools (https://surfer.nmr.mgh.harvard.edu/fswiki/QATools), resulting in the exclusion of additional 13 subjects. GWPC values were available for 91 VP/VLBW subjects and 105 FT subjects.

### 2.6 Statistical analysis

All statistical analyses were performed using IBM SPSS Version 26 (IBM Corp). Age was not included as a covariate in our analyses, as VP/VLBW subjects and FT controls had the same mean age of 26 years (*p* = 0.274). To control for possible scanner effects, we applied ComBat.^46^ ComBat‐harmonized values of the main outcome measure, GWPC at different projection fractions, and of CTh as a covariate of no interest were used for the analyses. We checked the main outcome measure, GWPC at different projection fractions, for outliers. As a criterion for outlier values, the interquartile range was multiplied by the factor 3. Additional five VP/VLBW and two FT subjects were excluded from analyses because they contained multiple outlier values. Finally, 86 VP/VLBW subjects and 103 FT subjects were included in the analyses. Normal distribution was assessed using histogram analysis and the Shapiro–Wilk test.

#### 2.6.1 Group comparison of GWPC

To analyze group differences between VP/VLBW and FT individuals, we used general linear models. GWPC values at different projection fractions (0%, 10%, 20%, 30%, 40%, 50%, and 60%) were entered as dependent variables. Group (VP/VLBW vs. FT) was entered as a fixed factor, and sex as a factor of no interest. The analyses at different projection fractions were false discovery rate (FDR) corrected for multiple comparisons across all 68 ROIs, respectively, using the Benjamini‐Hochberg procedure.^47^

As a control analysis for possible scanner effects, we repeated general linear model analyses for ROIs with significant differences between VP/VLBW individuals and FT controls using only data from one scanner, Munich Achieva 3 T (see Tables S2 and S3).

As a control analysis for possible confounding effects of lower CTh in VP/VLBW individuals, we repeated the general linear model analyses for ROIs with significant differences between VP/VLBW individuals and FT controls with CTh as an additional covariate of no interest (see Tables S4 and S5).

To investigate whether group differences in GWPC were specifically related to birth variables, we used two‐tailed partial correlation analyses within the VP/VLBW group. If group differences were identified, GWPC at the respective projection fractions in the respective ROIs was correlated with GA, BW, and duration of ventilation. Sex was entered as a covariate of no interest. Results at different projection fractions are reported at *p* < 0.05, uncorrected, and at *p* < 0.05, FDR‐corrected for multiple comparisons across all correlations regarding birth variables, respectively. And 95% confidence intervals for partial correlation analyses were obtained using a bootstrap approach (with 5000 repetitions) in SPSS.

#### 2.6.2 Relationship between GWPC and cognitive performance

To explore the relationship between altered GWPC after preterm birth and cognitive performance as measured by full‐scale IQ, we used two‐tailed partial correlation analyses within the VP/VLBW group. If group differences were found, GWPC at the respective projection fractions in the respective ROIs was correlated with full‐scale IQ. Sex was entered as a covariate of no interest. Results at different projection fractions are reported at *p* < 0.05, uncorrected, and at *p* < 0.05, FDR‐corrected for multiple comparisons across all seven correlations regarding cognitive performance, respectively. And 95% confidence intervals for partial correlation analyses were obtained using a bootstrap approach (with 5000 repetitions) in SPSS.

## 3 RESULTS

### 3.1 Sample characteristics

Table 1 presents group demographic and clinical background variables. There was no significant difference between the VP/VLBW group and FT group regarding sex (*p* = 0.736) and age at scanning (*p* = 0.247). By design of the study, VP/VLBW subjects had significantly lower GA (*p* < 0.001) and lower BW (*p* < 0.001). Furthermore, VP/VLBW subjects had significantly lower full‐scale IQ scores compared to FT controls (*p* < 0.001).

**TABLE 1:** Demographical, clinical, and cognitive data.

	VP/VLBW (*n* = 86)			FT (*n* = 103)			*p*‐Value
	Mean/*n*	SD	Range	Mean/*n*	SD	Range	
Sex (male/female)	48/38			60/43			0.736
Age (years)	26.8	± 0.6	25.7–28.3	26.8	± 0.7	25.5–28.9	0.247
GA (weeks)	30.6	± 2.2	25–36	39.7	± 1.1	37–42	<0.001*
BW (g)	1328	± 316	630–2000	3402	± 451	2120–4670	<0.001*
Ventilation (days)	11.4	± 17.1	0–81	n.a.	n.a.	n.a.	n.a.
SES	23/39/24		1–3	34/44/25		1–3	0.630
Full‐scale IQ (a.u.)^a^	94.0	± 12.8	64–131	102.3	± 11.9	77–130	<0.001*

*Note*: Statistical comparisons: sex and SES with *χ* ^2^ statistics; age, GA, BW, and full‐scale IQ with two sample *t*‐tests. Bold letters indicate statistical significance defined as *p* < 0.05. Asterisks (*) indicate statistical significance defined as *p* < 0.05, FDR‐corrected.

Abbreviations: BW, birth weight; FT, full‐term; GA, gestational age; IQ, intelligence quotient; n.a., not applicable; SD, standard deviation; SES, socioeconomic status at birth; VP/VLBW, very preterm and/or very low birthweight.

^a^ Data are based on 83 VP/VLBW individuals and 101 FT individuals.

### 3.2 Altered GWPC in preterm‐born adults

**FIGURE 1:**
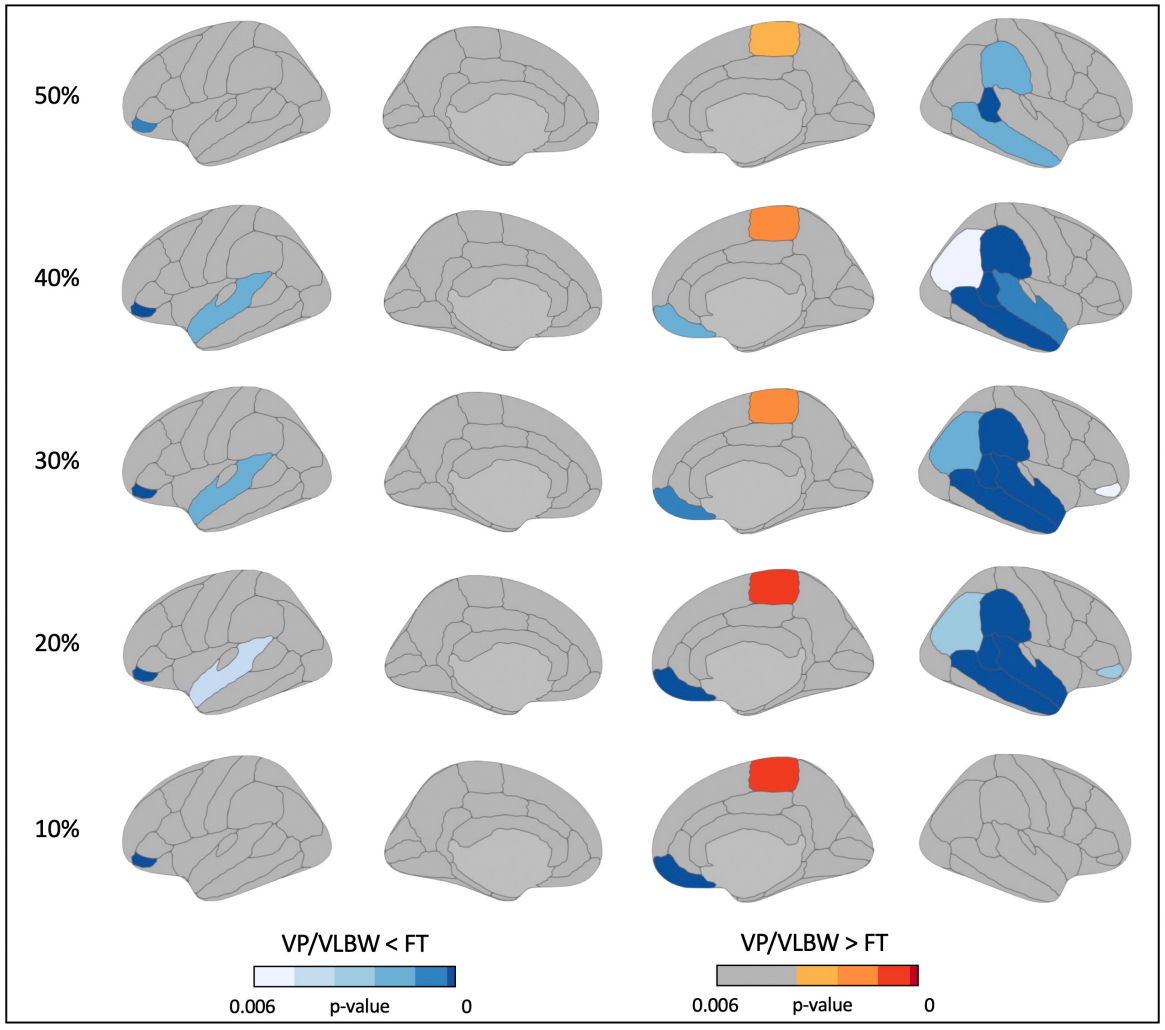
Group comparison of GWPC at 10%, 20%, 30%, 40%, and 50% projection fractions. All ROIs in which ComBat‐harmonized values of GWPC were significantly different in VP/VLBW individuals compared to FT controls. Statistical significance was defined as *p* < 0.05, FDR‐corrected. P‐values are color‐coded, darker colors indicate lower p‐values. Both hemispheres are shown in medial and lateral views. FT, full‐term; GWPC, gray‐to‐white matter percentage contrast; VP/VLBW, very preterm and/or very low birth weight.

GWPC was significantly (*p* < 0.05, FDR‐corrected) lower in associative cortices in the left inferior frontal and left superior temporal lobes, and in right frontal, parietal, and temporal lobes at 10%, 20%, 30%, 40%, and 50% projection fraction. Differences were more pronounced in the right hemisphere and at 20%, 30%, and 40% projection fraction. ROIs in which GWPC of VP/VLBW individuals was significantly lower compared to FT individuals with respective p‐values are listed in Table 2. In contrast, GWPC was significantly (*p* < 0.05, FDR‐corrected) higher in the right paracentral lobule (Table 3). Results are visualized in Figure 1.

**TABLE 2:** Group differences in GWPC for VP/VLBW<FT.

ROI	10% projection fraction			20% projection fraction			30% projection fraction			40% projection fraction			50% projection fraction		
	Estimated marginal mean		*p*‐Value	Estimated marginal mean		*p*‐Value	Estimated marginal mean		*p*‐Value	Estimated marginal mean		*p*‐Value	Estimated marginal mean		*p*‐Value
	VP/VLBW	FT		VP/VLBW	FT		VP/VLBW	FT		VP/VLBW	FT		VP/VLBW	FT	
Left hemisphere															
Parsorbitalis	11.254	11.694	<0.001*	13.907	14.567	<0.001*	15.942	16.714	<0.001*	17.454	18.253	<0.001*	18.745	19.542	0.001*
Superiortemporal	12.056	12.36	n.s.	14.755	15.176	0.004*	16.693	17.196	0.002*	18.029	18.557	0.002*	19.139	19.630	n.s.
Right hemisphere															
Bankssts	13.001	13.280	n.s.	15.883	16.347	<0.001*	18.050	18.620	<0.001*	19.582	20.177	<0.001*	20.796	21.350	<0.001*
Inferiorparietal	11.158	11.350	n.s.	13.454	13.778	0.003*	15.296	15.689	0.002*	16.781	17.159	0.005*	18.170	18.460	n.s.
Medialorbitofrontal	11.487	11.895	<0.001*	14.608	15.049	<0.001*	17.116	17.565	0.001*	19.001	19.436	0.002*	20.471	20.880	n.s.
Middletemporal	12.674	12.913	n.s.	15.636	16.074	<0.001*	17.801	18.349	<0.001*	19.264	19.823	<0.001*	20.354	20.853	0.002*
Parsorbitalis	11.576	11.881	n.s.	14.323	14.761	0.003*	16.458	16.926	0.005*	18.055	18.478	n.s.	19.389	19.758	n.s.
Superiortemporal	11.764	12.170	n.s.	14.524	15.075	<0.001*	16.519	17.161	<0.001*	17.884	18.538	0.001*	18.982	19.579	n.s.
Supramarginal	11.452	11.669	n.s.	13.906	14.294	<0.001*	15.811	16.303	<0.001*	17.262	17.761	<0.001*	18.546	18.970	0.002*

*Note*: ROIs in which GWPC of VP/VLBW individuals was significantly (*p* < 0.05, FDR corrected) lower compared to FT individuals with respective estimated marginal means and *p*‐values. Bold letters indicate statistical significance defined as *p* < 0.05. Asterisks (*) indicate statistical significance defined as *p* < 0.05, FDR‐corrected.

Abbreviations: Bankssts, Banks of the superior temporal sulcus; FT, full‐term; inferiorparietal, Inferior parietal cortex; medialorbitofrontal, Medial orbitofrontal cortex; middletemporal, Middle temporal gyrus; n.s., not significant; parsorbitalis, Inferior frontal gyrus, pars orbitalis; ROI, region of interest; superiortemporal, Superior temporal gyrus; supramarginal, Supramarginal gyrus; VP/VLBW, very preterm and/or very low birthweight.

**TABLE 3:** Group difference in GWPC for VP/VLBW>FT.

ROI	10% projection fraction			20% projection fraction			30% projection fraction			40% projection fraction			50% projection fraction		
	Estimated marginal mean		*p*‐Value	Estimated marginal mean		*p*‐Value	Estimated marginal mean		*p*‐Value	Estimated marginal mean		*p*‐Value	Estimated marginal mean		*p*‐Value
	VP/VLBW	FT		VP/VLBW	FT		VP/VLBW	FT		VP/VLBW	FT		VP/VLBW	FT	
Right hemisphere															
Paracentral	9.099	8.74	0.001*	10.975	10.567	0.001*	12.616	12.171	0.002*	14.092	13.617	0.002*	15.600	15.107	0.003*

*Note*: ROI in which GWPC of VP/VLBW individuals was significantly (*p* < 0.05, FDR corrected) higher compared to FT individuals with respective estimated marginal means and *p*‐values. Bold letters indicate statistical significance defined as *p* < 0.05. Asterisks (*) indicate statistical significance defined as *p* < 0.05, FDR‐corrected.

Abbreviations: FT, full‐term; paracentral, Paracentral lobule; ROI, region of interest; VP/VLBW, very preterm and/or very low birthweight.

As a control analysis for possible scanner effects, we repeated general linear model analyses for ROIs with significant differences at 10%, 20%, 30%, 40%, and 50% projection fraction between VP/VLBW individuals and FT controls using only data from one scanner (Munich Achieva 3 T). Except for two results in right inferior frontal gyrus, pars orbitalis, group differences in GWPC remained significant (p < 0.05, FDR‐corrected) using only data from one scanner (see Table S2 for GWPC in VP/VLBW<FT and Table S3 for GWPC in VP/VLBW>FT).

To control for possible confounding effects of lower CTh in VP/VLBW individuals, we repeated general linear model analyses for ROIs with significant differences at 10%, 20%, 30%, 40%, and 50% projection fraction between VP/VLBW individuals and FT controls with CTh as a covariate of no interest. Except for few results in right inferior parietal cortex and right inferior frontal gyrus, pars orbitalis, group differences in GWPC remained significant (*p* < 0.05, FDR‐corrected) when additionally controlling for CTh (see Table S4 for GWPC in VP/VLBW<FT and Table S5 for GWPC in VP/VLBW>FT).

**FIGURE 2:**
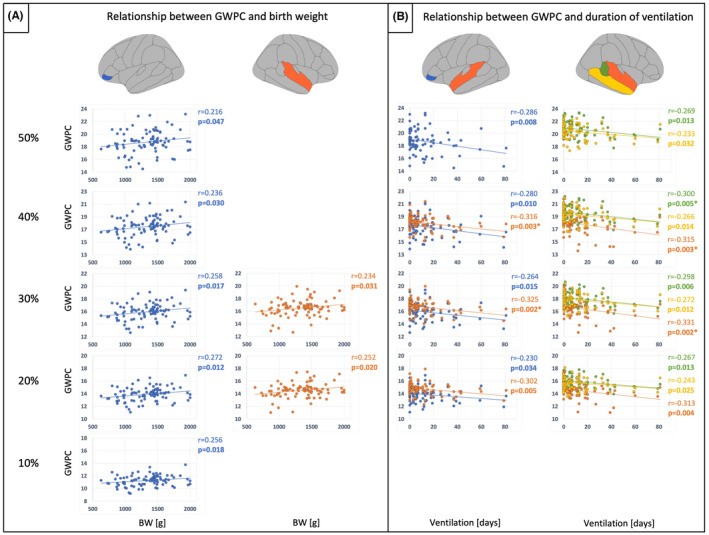
Relationship between GWPC and birth variables. (A) Scatter plots showing significant (*p* < 0.05) results of partial correlation analyses between GWPC and BW for GWPC in VP/VLBW<FT. BW (in grams) is plotted on the x‐axes and GWPC is plotted on the y‐axes. (B) Scatter plots showing significant (*p* < 0.05) results of partial correlation analyses between GWPC and duration of ventilation for GWPC in VP/VLBW<FT. Duration of ventilation (in days) is plotted on the x‐axes and GWPC is plotted on the y‐axes. Linear regression lines as well as correlation coefficients and p‐values were added. Bold letters indicate statistical significance defined as *p* < 0.05. Asterisks (*) indicate statistical significance defined as *p* < 0.05, FDR‐corrected. BW, birth weight; GWPC, gray‐to‐white matter percentage contrast

To test whether group differences in GWPC are specifically related to preterm birth, we performed partial correlation analyses between GWPC in ROIs with significant differences at 10%, 20%, 30%, 40%, and 50% projection fraction and GA, BW, and duration of ventilation. GWPC in the left inferior frontal gyrus, pars orbitalis, and right superior temporal gyrus was positively correlated with BW, and GWPC in the left inferior frontal gyrus, pars orbitalis, left superior temporal gyrus, right banks of the superior temporal sulcus, right middle temporal gyrus, and right superior temporal gyrus was negatively correlated with duration of ventilation (*p* < 0.05, respectively). However, only the associations between the duration of ventilation and left and right superior temporal gyrus at 30% and 40% projection fraction and between the duration of ventilation and right banks of the superior temporal sulcus at 40% projection fraction remained significant after FDR‐correction. Significant (*p* < 0.05) results are listed in Table 4 and visualized as scatter plots in Figure 2. All results are listed in Tables S6 and S7.

**TABLE 4:** Relationship between GWPC and birth variables for GWPC in VP/VLBW<FT.

Birth variable	ROI	10% projection fraction				20% projection fraction				30% projection fraction				40% projection fraction				50% projection fraction			
		*r*	95% CI		*p*‐Value	*r*	95% CI		*p*‐Value	*r*	95% CI		*p*‐Value	*r*	95% CI		*p*‐Value	*r*	95% CI		*p*‐Value
GA	n.s.																				
BW	Left hemisphere																				
	Parsorbitalis	0.256	0.050	0.451	**0.018**	0.272	0.063	0.467	**0.012**	0.258	0.043	0.455	**0.017**	0.236	0.026	0.444	**0.030**	0.216	0.008	0.414	**0.047**
	Right hemisphere																				
	Superiortemporal	n.a.	n.a.	n.a.	n.a.	0.252	0.062	0.425	**0.020**	0.234	0.044	0.410	**0.031**	n.s.	n.s.	n.s.	n.s.	n.a.	n.a.	n.a.	n.a.
Ventilation	Left hemisphere																				
	Parsorbitalis	n.s.	n.s.	n.s.	n.s.	−0.230	−0.426	−0.018	**0.034**	−0.264	−0.459	−0.047	**0.015**	−0.280	−0.467	−0.059	**0.010**	−0.286	−0.476	−0.061	**0.008**
	Superiortemporal	n.a.	n.a.	n.a.	n.a.	−0.302	−0.478	−0.123	**0.005**	−0.325	−0.506	−0.143	0.002*	−0.316	−0.492	−0.135	0.003*	n.a.	n.a.	n.a.	n.a.
	Right hemisphere																				
	Bankssts	n.a.	n.a.	n.a.	n.a.	−0.267	−0.419	−0.089	**0.013**	−0.298	−0.458	−0.107	**0.006**	−0.300	−0.462	−0.111	0.005*	−0.269	−0.437	−0.076	**0.013**
	Middletemporal	n.a.	n.a.	n.a.	n.a.	−0.243	−0.435	−0.028	**0.025**	−0.272	−0.467	−0.061	**0.012**	−0.266	−0.456	−0.049	**0.014**	−0.233	−0.425	−0.023	**0.032**
	Superiortemporal	n.a.	n.a.	n.a.	n.a.	−0.313	−0.482	−0.154	**0.004**	−0.331	−0.509	−0.162	0.002*	−0.315	−0.500	−0.132	0.003*	n.a.	n.a.	n.a.	n.a.

*Note*: Significant (*p* < 0.05) results of partial correlation analyses between GWPC and GA, BW, and duration of ventilation with correlation coefficients, 95% confidence intervals, and *p*‐values. Bold letters indicate statistical significance defined as *p* < 0.05. Asterisks (*) indicates statistical significance defined as *p* < 0.05, FDR‐corrected.

Abbreviations: Bankssts, Banks of the superior temporal sulcus; BW, birth weight; GA, gestational age; inferiorparietal, Inferior parietal cortex; medialorbitofrontal, Medial orbitofrontal cortex; middletemporal, Middle temporal gyrus; n.a., not applicable; n.s., not significant; parsorbitalis, Inferior frontal gyrus, pars orbitalis; ROI, region of interest; superiortemporal, Superior temporal gyrus; supramarginal, Supramarginal gyrus.

### 3.3 Functional relevance of lower GWPC

**FIGURE 3:**
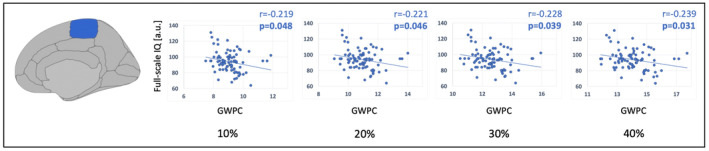
Relationship between GWPC and cognitive performance. Scatter plots showing significant (*p* < 0.05) results of partial correlation analyses between GWPC and full‐scale IQ for GWPC in VP/VLBW>FT. GWPC is plotted on the x‐axes and full‐scale IQ is plotted on the Y‐axes. GWPC, gray‐to‐white matter percentage contrast.

To explore the functional relevance of lower GWPC, we performed partial correlation analyses between GWPC in ROIs with significant differences at 10%, 20%, 30%, 40%, and 50% projection fraction and full‐scale IQ. GWPC in the right paracentral lobule was negatively correlated with full‐scale IQ at 10%, 20%, 30%, and 40% projection fraction (*p* < 0.05). However, results did not remain significant after FDR correction. All significant (*p* < 0.05) results are listed in Table 5 and visualized as scatter plots in Figure 3. All results are listed in Tables S8 and S9.

**TABLE 5:** Relationship between GWPC and cognitive performance for GWPC in VP/VLBW>FT.

ROI	Cognitive performance	10% projection fraction				20% projection fraction				30% projection fraction				40% projection fraction				50% projection fraction			
		*r*	95% CI		*p*‐Value	*r*	95% CI		*p*‐Value	*r*	95% CI		*p*‐Value	*r*	95% CI		*p*‐Value	*r*	95% CI		*p*‐Value
Right hemisphere	Full‐scale IQ																				
Paracentral		−0.219	−0.445	0.009	**0.048**	−0.221	−0.434	0.015	**0.046**	−0.228	−0.447	0.004	**0.039**	−0.239	−0.451	−0.004	**0.031**	n.s.	n.s.	n.s.	n.s.

*Note*: Significant (*p* < 0.05) results of partial correlation analyses between GWPC in the right paracentral lobule and full‐scale IQ with correlation coefficients, 95% confidence intervals, and *p*‐values. Bold letters indicate statistical significance defined as *p* < 0.05. Asterisks (*) indicate statistical significance defined as *p* < 0.05, FDR‐corrected.

Abbreviations: IQ, intelligence quotient; n.a., not applicable; n.s., not significant; paracentral, Paracentral lobule; ROI, region of interest.

## 4 DISCUSSION

Using structural MRI, we found significantly lower tissue contrast between gray matter within the cortex and white matter in preterm‐born adults compared to controls in frontal, parietal, and temporal associative cortices, predominantly in the right hemisphere, at 10%, 20%, 30%, 40%, and 50% projection fraction. Differences were more pronounced at 20%, 30%, and 40%, hence, in middle cortical layers. Frontal and temporal GWPC was positively correlated with BW and negatively correlated with duration of ventilation. In contrast, GWPC in the right paracentral lobule was significantly higher in preterm‐born adults compared to controls. Furthermore, GWPC in the right paracentral lobule was negatively correlated with full‐scale IQ, indicating that altered GWPC might contribute to lasting cognitive deficits after preterm birth. To the best of our knowledge, results provide first evidence for widespread aberrant gray‐to‐white matter tissue contrast after preterm birth. Data suggest regionally altered cortical microstructure in human preterm‐born adults, mainly in middle cortical layers, with differential effects on associative and primary cortices.

### 4.1 Altered gray‐to‐whiter matter contrast after preterm birth

We found lower GWPC in VP/VLBW adults compared to FT controls in frontal, parietal, and temporal associative cortices predominantly in the right hemisphere and mainly at 20%, 30%, and 40% projection fraction, indicating lower tissue contrast between gray matter within the cortex and white matter. Frontal and temporal GWPC was correlated with BW and duration of ventilation. In contrast, we found higher GWPC in VP/VLBW adults compared to FT controls in the right paracentral lobule at 10%, 20%, 30%, 40%, and 50% projection fraction, indicating higher tissue contrast between gray matter within the cortex and white matter in primary cortices.

Previous results in healthy subjects, in patients with Alzheimer's disease, in patients with schizophrenia, and in patients with autism spectrum disorder suggest lower contrast in aging and age‐associated disease as well as in neurodevelopmental and psychiatric disorders that are associated with abnormal SPN development.^14^, ^15^, ^16^, ^17^, ^18^, ^19^, ^48^ More specifically, Salat et al.^15^ reported that gray‐to‐white matter contrast at 35% projection fraction decreased with age. Furthermore, gray‐to‐white matter contrast at 35% projection fraction was decreased in individuals with Alzheimer's disease compared to non‐demented control participants.^16^ Therefore, widespread lower GWPC in preterm‐born adults compared to controls could be interpreted as accelerated brain aging, which is in line with our previous results suggesting an increased risk for accelerated brain aging in human prematurity.^20^ However, results have to be interpreted with care since cross‐sectional data cannot answer questions regarding brain development. Longitudinal studies across different age groups are necessary to investigate brain aging after preterm birth.

On a cellular level, altered gray‐to‐white‐matter contrast might be associated with abnormal SPN development. Decreased gray‐to‐white matter contrast was reported in patients with schizophrenia at 35% projection fraction and in individuals with autism spectrum disorder at all sampling depths from 0% (gray/white matter boundary) to 60%.^14^, ^17^ More specifically, Andrews et al.^14^ found that reductions in GWPC were most extensive when gray matter intensity was sampled at the gray/white matter boundary. These in‐vivo results are in line with previous postmortem histological studies that report abnormal cell patterning at the cortical gray/white matter boundary in autism spectrum disorders.^49^ Possible explanations for this less distinct boundary include disrupted migratory processes or improper resolution of the cortical subplate.^49^ In contrast, in the present study, we found differences in GWPC between preterm‐born adults and controls predominantly at 20%, 30%, and 40% projection fraction, while GWPC was not significantly altered at the gray/white matter boundary. Hence, we found no evidence for abnormal persistence of SPN at the gray/white matter boundary after preterm birth. However, lower contrast at projection fractions within the cortex could still reflect SPN dysmaturation. SPNs are particularly important for the correct development of thalamocortical connections as they provide a scaffold for thalamic inputs to directly innervate cortical layer 4.^12^, ^50^, ^51^ The relative thickness of cortical layers in humans indicates that projection fractions between 30% and 40% represent layer 4.^52^, ^53^ Therefore, our results could indicate alterations in thalamocortical projections after preterm birth. In line with this interpretation, we recently found decreased connection probability between bilateral temporal cortices and bilateral anterior thalami using diffusion‐weighted imaging in the same cohort of preterm‐born adults.^54^

Another possible explanation for differences in GWPC could also be abnormal pre‐OL development. Besides SPNs, pre‐OLs are particularly vulnerable to hypoxia‐ischemia.^8^, ^9^ More specifically, the maturation of pre‐OLs to myelin‐producing OLs is impaired after premature birth, leading to disturbed myelination.^8^, ^55^ Since the cortex also contains myelinated axons, altered cortical myelination could contribute to differences in GWPC between preterm‐born adults and controls.

Importantly, our results suggest differential effects of prematurity on the microstructural organization of associative and primary cortices. Diverse neurobiological properties reveal a sensorimotor‐to‐association axis of cortical organization, such as cortical myelination and thickness.^56^, ^57^ It has been suggested that protracted plasticity within late‐maturing association cortices makes these cortical areas particularly vulnerable to the effects of developmental insults,^56^ which is in line with our findings of widespread alterations in associative cortices.

Furthermore, we previously investigated cortical architecture after preterm birth using CTh.^23^ We found lower CTh in frontal, parietal, and temporal associative cortices predominantly in the left hemisphere in premature‐born adults compared to controls.^23^ However, there was only very little spatial overlap (left and right inferior frontal gyrus, pars orbitalis) between ROIs with lower CTh and ROIs with aberrant GWPC after preterm birth. Moreover, most of our results remained significant after controlling for CTh (see Tables S2 and S3). Hence, GWPC seems to describe structural cortical aberrations after preterm birth beyond alterations in CTh, which is in line with findings in individuals with Alzheimer's disease, schizophrenia, and autism spectrum disorder.^14^, ^16^, ^17^

### 4.2 Functional relevance of altered gray‐to‐white matter contrast

GWPC in the right paracentral lobule was negatively correlated with full‐scale IQ (*p* < 0.05), indicating that altered GWPC might contribute to lasting cognitive deficits after preterm birth.

While the association between gray‐to‐white matter contrast and cognitive performance has not been investigated in healthy adults or after preterm birth, previous results in individuals with mild cognitive impairment and Alzheimer's disease suggest that lower contrast in patients compared to controls is associated with cognitive decline.^16^, ^58^, ^59^ In this sample of preterm‐born adults, GWPC in the right paracentral lobule was higher compared to controls; hence, the negative correlation with IQ might still indicate that altered GWPC contributes to lasting cognitive deficits after preterm birth. Furthermore, the paracentral lobule is part of the sensorimotor network, which has been associated with cognitive impairment using functional MRI.^60^ However, our results did not remain significant after FDR correction. Hence, on the other hand, it is possible that GWPC does not reflect cortical features that are important for cognitive performance. In general, analyses trying to link brain structure with cognitive functioning have to be interpreted with care, as only one specific aspect is investigated, while multiple other structural features have been associated with cognitive performance.^3^, ^21^, ^22^, ^23^, ^61^ More specifically, other features of cortical architecture, such as gyrification and CTh, have been linked with full‐scale IQ in preterm‐born adults.^21^, ^22^, ^23^ Furthermore, individual, social, and environmental factors influence the association between brain structural features and cognitive performance.

### 4.3 Strengths and limitations

One important limitation of the current study is the resolution of structural MRI images (1 mm isotropic voxels). At this level of resolution that neuroimaging techniques currently offer, it is not possible to distinguish between different aspects of cortical cytoarchitecture or accurately delineate particular layers of the cortical sheet as defined by histological staining.^14^

Furthermore, the current sample is biased toward VP/VLBW adults with less severe neonatal complications, less functional impairments, and higher IQ. Individuals with more birth complications in the initial BLS sample were more likely to be excluded due to exclusion criteria for MRI. Thus, the reported differences in GWPC between VP/VLBW and FT controls are conservative estimates of true differences. However, in terms of GA and BW, our final sample was still representative of the full cohort since these values were not significantly different in VP/VLBW subjects with MRI data compared to subjects without MRI data (see Table S10).

One of the strengths of our study is that a relevant impact of age is controlled because VP/VLBW subjects and FT controls had the same age of 26 years at the time of the MRI scan.

Furthermore, the study has the strength of a large sample (86 VP/VLBW and 103 FT adults).

## 5 CONCLUSION

Tissue contrast between gray matter within the cortex and white matter is lower in preterm‐born adults compared to controls. Our results indicate lastingly altered cortical microstructure after preterm birth with differential effects on associative and primary cortices.

## FUNDING INFORMATION

This work was supported by the Deutsche Forschungsgemeinschaft (SO 1336/1‐1 to C.S., HE 8967/3‐1 to D.M.H., and ME 5894/2‐1 to A.M.), German Federal Ministry of Education and Science (BMBF 01ER0801 to P.B. and D.W., BMBF 01ER0803 to C.S.), the RECAP preterm project, an EU Horizon 2020 study (supported by grant 733280; to D.W. and P.B.), the UKRI Frontier Research Grant EP/X023206/1 (ERC‐AdG reviewed; to D.W.) and the Kommission für Klinische Forschung, Technische Universität München (KKF 8765162 to C.S., KKF8700000474 to D.M.H., and KKF 8700000620 to B.S.‐K).

## CONFLICT OF INTEREST STATEMENT

No potential conflicts of interest.

## Supporting information


Appendix S1

## Data Availability

Patient data used in this study are not publicly available but stored by the principal investigators of the Bavarian Longitudinal Study.
