# Supplementary material for: Altered gray‐to‐white matter tissue contrast in preterm‐born adults
Source: CNS Neurosci Ther. 2023 Jun 27;29(11):3199–211. doi: 10.1111/cns.14320 (PMC10580354; doi:10.1111/cns.14320)
Supplement: Supplementary file 1 — Appendix S1 [file CNS-29-3199-s001.docx]

**- Supplement-**

**S1: Participants – Bavarian Longitudinal Study**

All subjects were part of the Bavarian Longitudinal Study (BLS), a geographically defined, whole-population sample of neonatal at-risk children and healthy FT controls who were followed from birth, between January 1985 and March 1986, into adulthood.^1,2^ 682 infants were born VP (<32 weeks of gestation) and/or with very low birth weight (VLBW, birth weight <1500g). Informed written consent from a parent and/or legal guardian was obtained. From the initial 916 FT born infants born at the same obstetric hospitals who were alive at 6 years, 350 were randomly selected as control subjects within the stratification variables of sex and family socioeconomic status in order to be comparable with the VP/VLBW sample. Of these, 411 VP/VLBW individuals and 308 controls were eligible for the 26-year follow-up assessment. 260 of the VP/VLBW group and 229 controls participated in psychological assessments.^3^ All subjects were screened for MR-related exclusion criteria, including (self-reported): claustrophobia, inability to lie still for >30 minutes, unstable medical conditions (e.g., severe asthma), epilepsy, tinnitus, pregnancy, non-removable MRI-incompatible metal implants, and a history of severe CNS trauma or disease that would impair further analysis of the data. However, the most frequent reason not to perform the MRI exam was that subjects declined to participate.

**Table S2: Group differences in GWPC for VP/VLBW<FT using data from Munich Achieva 3T**

| **ROI** | **10% projection fraction** | | **20% projection fraction** | | **30% projection fraction** | | **40% projection fraction** | | **50% projection fraction** | |
| --- | --- | --- | --- | --- | --- | --- | --- | --- | --- | --- |
|  | **All scanners**  **p-value** | **Munich Achieva 3T**  **p-value** | **All scanners**  **p-value** | **Munich Achieva 3T**  **p-value** | **All scanners**  **p-value** | **Munich Achieva 3T**  **p-value** | **All scanners**  **p-value** | **Munich Achieva 3T**  **p-value** | **All scanners**  **p-value** | **Munich Achieva 3T**  **p-value** |
| **Left hemisphere**  parsorbitalis  superiortemporal  **Right hemisphere**  bankssts  inferiorparietal  medialorbitofrontal  middletemporal  parsorbitalis  superiortemporal  supramarginal | **<0.001***  n.s.  n.s.  n.s.  **<0.001***  n.s.  n.s.  n.s.  n.s. | **0.004***  n.a.  n.a.  n.a.  **0.014***  n.a.  n.a.  n.a.  n.a. | **<0.001***  **0.004***  **<0.001***  **0.003***  **<0.001***  **<0.001***  **0.003***  **<0.001***  **<0.001*** | **<0.001***  **0.031***  **0.002***  **0.015***  **0.012***  **0.008***  0.091  **0.024***  **<0.001*** | **<0.001***  **0.002***  **<0.001***  **0.002***  **0.001***  **<0.001***  **0.005***  **<0.001***  **<0.001*** | **<0.001***  **0.013***  **0.001***  **0.010***  **0.014***  **0.003***  0.103  **0.015***  **<0.001*** | **<0.001***  **0.002***  **<0.001***  **0.005***  **0.002***  **<0.001***  n.s**.**  **0.001***  **<0.001*** | **0.001***  **0.012***  **0.002***  **0.020***  **0.023***  **0.005***  n.a**.**  **0.023***  **<0.001*** | **0.001***  n.s.  **<0.001***  n.s.  n.s.  **0.002***  n.s**.**  n.s.  **0.002*** | **0.005***  n.a.  **0.008***  n.a.  n.a.  **0.016***  n.a**.**  n.a.  **0.002*** |

ROIs in which GWPC of VP/VLBW individuals was significantly lower compared to FT individuals using data from all scanners and only data from Munich Achieva 3T with the respective p-values. Bold letters indicate statistical significance defined as p<0.05. Asterisks (*) indicate statistical significance defined as p<0.05, FDR-corrected.

Abbreviations: Bankssts, Banks of the superior temporal sulcus; inferiorparietal, Inferior parietal cortex; medialorbitofrontal, Medial orbitofrontal cortex; middletemporal, Middle temporal gyrus; n.a., not applicable; n.s., not significant; parsorbitalis, Inferior frontal gyrus, pars orbitalis; ROI, region of interest; superiortemporal, Superior temporal gyrus; supramarginal, Supramarginal gyrus.

**Table S3: Group differences in GWPC for VP/VLBW>** **FT using data from Munich Achieva 3T**

| **ROI** | **10% projection fraction** | | **20% projection fraction** | | **30% projection fraction** | | **40% projection fraction** | | **50% projection fraction** | |
| --- | --- | --- | --- | --- | --- | --- | --- | --- | --- | --- |
|  | **All scanners**  **p-value** | **Munich Achieva 3T**  **p-value** | **All scanners**  **p-value** | **Munich Achieva 3T**  **p-value** | **All scanners**  **p-value** | **Munich Achieva 3T**  **p-value** | **All scanners**  **p-value** | **Munich Achieva 3T**  **p-value** | **All scanners**  **p-value** | **Munich Achieva 3T**  **p-value** |
| **Right hemisphere**  paracentral | **0.001*** | **0.011*** | **0.001*** | **0.016*** | **0.002*** | **0.022*** | **0.002*** | **0.030*** | **0.003*** | **0.041*** |

ROI in which GWPC of VP/VLBW individuals was significantly higher compared to FT individuals using data from all scanners and only data from Munich Achieva 3T with the respective p-values. Bold letters indicate statistical significance defined as p<0.05. * indicates statistical significance defined as p<0.05, FDR-corrected.

Abbreviations: paracentral, Paracentral lobule; ROI, region of interest.

**Table S4: Group differences in GWPC for VP/VLBW<FT controlling for cortical thickness**

| **ROI** | **10% projection fraction** | | **20% projection fraction** | | **30% projection fraction** | | **40% projection fraction** | | **50% projection fraction** | |
| --- | --- | --- | --- | --- | --- | --- | --- | --- | --- | --- |
|  | **p-value** | **With CTh as covariate**  **p-value** | **p-value** | **With CTh as covariate**  **p-value** | **p-value** | **With CTh as covariate**  **p-value** | **p-value** | **With CTh as covariate**  **p-value** | **p-value** | **With CTh as covariate**  **p-value** |
| **Left hemisphere**  parsorbitalis  superiortemporal  **Right hemisphere**  bankssts  inferiorparietal  medialorbitofrontal  middletemporal  parsorbitalis  superiortemporal  supramarginal | **<0.001***  n.s.  n.s.  n.s.  **<0.001***  n.s.  n.s.  n.s.  n.s. | **0.007***  n.a.  n.a.  n.a.  **<0.001***  n.a.  n.a.  n.a.  n.a. | **<0.001***  **0.004***  **<0.001***  **0.003***  **<0.001***  **<0.001***  **0.003***  **<0.001***  **<0.001*** | **0.005***  **0.003***  **0.010***  0.052  **<0.001***  **0.026***  0.097  **0.002***  **0.012*** | **<0.001***  **0.002***  **<0.001***  **0.002***  **0.001***  **<0.001***  **0.005***  **<0.001***  **<0.001*** | **0.008***  **0.001***  **0.005***  **0.043***  **<0.001***  **0.012***  0.154  **0.001***  **<0.001*** | **<0.001***  **0.002***  **<0.001***  **0.005***  **0.002***  **<0.001***  n.s**.**  **0.001***  **<0.001*** | **0.020***  **0.002***  **0.005***  0.054  **<0.001***  **0.013***  n.a**.**  **0.002***  **0.005*** | **0.001***  n.s.  **<0.001***  n.s.  n.s.  **0.002***  n.s**.**  n.s.  **0.002*** | **0.042***  n.a.  **0.011***  n.a.  n.a.  **0.025***  n.a**.**  n.a.  **0.013*** |

ROIs in which GWPC of VP/VLBW individuals was significantly lower compared to FT individuals without and with CTh as a covariate with the respective p-values. Bold letters indicate statistical significance defined as p<0.05. Asterisks (*) indicate statistical significance defined as p<0.05, FDR-corrected.

Abbreviations: Bankssts, Banks of the superior temporal sulcus; CTh, cortical thickness; inferiorparietal, Inferior parietal cortex; medialorbitofrontal, Medial orbitofrontal cortex; middletemporal, Middle temporal gyrus; n.a., not applicable; n.s., not significant; parsorbitalis, Inferior frontal gyrus, pars orbitalis; ROI, region of interest; superiortemporal, Superior temporal gyrus; supramarginal, Supramarginal gyrus.

**Table S5: Group differences in GWPC for VP/VLBW>FT controlling for cortical thickness**

| **ROI** | **10% projection fraction** | | **20% projection fraction** | | **30% projection fraction** | | **40% projection fraction** | | **50% projection fraction** | |
| --- | --- | --- | --- | --- | --- | --- | --- | --- | --- | --- |
|  | **p-value** | **With CTh as covariate**  **p-value** | **p-value** | **With CTh as covariate**  **p-value** | **p-value** | **With CTh as covariate**  **p-value** | **p-value** | **With CTh as covariate**  **p-value** | **p-value** | **With CTh as covariate**  **p-value** |
| **Right hemisphere**  paracentral | **0.001*** | **0.002*** | **0.001*** | **0.002*** | **0.002*** | **0.002*** | **0.002*** | **0.002*** | **0.003*** | **0.002*** |

ROI in which GWPC of VP/VLBW individuals was significantly higher compared to FT individuals without and with CTh as covariate with the respective p-values. Bold letters indicate statistical significance defined as p<0.05. * indicates statistical significance defined as p<0.05, FDR-corrected.

Abbreviations: CTh, cortical thickness; paracentral, Paracentral lobule; ROI, region of interest.

**Table S6: Relationship between GWPC and birth variables for GWPC in VP/VLBW<FT**

| **Birth variable** | **ROI** | **10% projection fraction** | | | | **20% projection fraction** | | | | **30% projection fraction** | | | | **40% projection fraction** | | | | **50% projection fraction** | | | |
| --- | --- | --- | --- | --- | --- | --- | --- | --- | --- | --- | --- | --- | --- | --- | --- | --- | --- | --- | --- | --- | --- |
|  |  | **r** | **95% CI** | | **p-value** | **r** | **95% CI** | | **p-value** | **r** | **95% CI** | | **p-value** | **r** | **95% CI** | | **p-value** | **r** | **95% CI** | | **p-value** |
| GA | **Left hemisphere**  parsorbitalis  superiortemporal  **Right hemisphere**  bankssts  inferiorparietal  medialorbitofrontal  middletemporal  parsorbitalis  superiortemporal  supramarginal | 0.071  n.a.  n.a.  n.a.  0.052  n.a.  n.a.  n.a.  n.a. | -0.084  n.a.  n.a.  n.a.  -0.125  n.a.  n.a.  n.a.  n.a. | 0.227  n.a.  n.a.  n.a.  0.238  n.a.  n.a.  n.a.  n.a. | 0.517  n.a.  n.a.  n.a.  0.636  n.a.  n.a.  n.a.  n.a. | 0.109  0.087  0.110  0.094  0.028  0.199  -0.113  0.184  0.024 | -0.058  -0.077  -0.061  -0.097  -0.166  0.017  -0.334  0.031  -0.161 | 0.265  0.257  0.279  0.278  0.227  0.368  0.110  0.334  0.201 | 0.321  0.426  0.318  0.393  0.798  0.068  0.305  0.092  0.828 | 0.126  0.104  0.128  0.113  0.017  0.209  -0.088  0.188  0.042 | -0.036  -0.080  -0.048  -0.085  -0.190  0.022  -0.309  0.034  -0.142 | 0.290  0.289  0.307  0.306  0.224  0.380  0.136  0.348  0.221 | 0.251  0.345  0.244  0.304  0.880  0.055  0.423  0.084  0.702 | 0.129  0.103  0.126  0.099  0.017  0.200  n.a.  0.176  0.036 | -0.034  -0.083  -0.058  -0.115  -0.184  0.014  n.a.  0.009  -0.147 | 0.296  0.283  0.309  0.304  0.231  0.377  n.a.  0.340  0.222 | 0.238  0.349  0.251  0.365  0.874  0.067  n.a.  0.107  0.745 | 0.129  n.a.  0.105  n.a.  n.a.  0.181  n.a.  n.a.  0.019 | -0.039  n.a.  -0.083  n.a.  n.a.  -0.003  n.a.  n.a.  -0.158 | 0.298  n.a.  0.286  n.a.  n.a.  0.356  n.a.  n.a.  0.191 | 0.240  n.a.  0.338  n.a.  n.a.  0.097  n.a.  n.a.  0.865 |
| BW | **Left hemisphere**  parsorbitalis  superiortemporal  **Right hemisphere**  bankssts  inferiorparietal  medialorbitofrontal  middletemporal  parsorbitalis  superiortemporal  supramarginal | 0.256  n.a.  n.a.  n.a.  0.129  n.a.  n.a.  n.a.  n.a. | 0.050  n.a.  n.a.  n.a.  -0.061  n.a.  n.a.  n.a.  n.a. | 0.451  n.a.  n.a.  n.a.  0.322  n.a.  n.a.  n.a.  n.a. | **0.018**  n.a.  n.a.  n.a.  0.241  n.a.  n.a.  n.a.  n.a. | 0.272  0.201  0.065  -0.048  0.098  0.004  0.156  0.252  -0.028 | 0.063  -0.025  -0.151  -0.266  -0.116  -0.214  -0.042  0.062  -0.236 | 0.467  0.409  0.283  0.184  0.306  0.222  0.355  0.425  0.194 | **0.012**  0.065  0.554  0.664  0.372  0.974  0.155  **0.020**  0.802 | 0.258  0.196  0.063  -0.066  0.076  -0.01  0.150  0.234  -0.037 | 0.043  -0.031  -0.150  -0.279  -0.155  -0.216  -0.052  0.044  -0.234 | 0.455  0.402  0.272  0.154  0.291  0.202  0.345  0.410  0.178 | **0.017**  0.073  0.565  0.547  0.491  0.928  0.171  **0.031**  0.738 | 0.236  0.180  0.048  -0.084  0.069  -0.029  n.a.  0.201  -0.063 | 0.026  -0.045  -0.164  -0.287  -0.166  -0.239  n.a.  -0.001  -0.257 | 0.444  0.397  0.263  0.148  0.292  0.189  n.a.  0.386  0.151 | **0.030**  0.099  0.666  0.445  0.530  0.791  n.a.  0.065  0.569 | 0.216  n.a.  0.024  n.a.  n.a.  -0.048  n.a.  n.a.  -0.093 | 0.008  n.a.  -0.190  n.a.  n.a.  -0.258  n.a.  n.a.  -0.293 | 0.414  n.a.  0.234  n.a.  n.a.  0.171  n.a.  n.a.  0.109 | **0.047**  n.a.  0.825  n.a.  n.a.  0.663  n.a.  n.a.  0.398 |
| Ventilation | **Left hemisphere**  parsorbitalis  superiortemporal  **Right hemisphere**  bankssts  inferiorparietal  medialorbitofrontal  middletemporal  parsorbitalis  superiortemporal  supramarginal | -0.162  n.a.  n.a.  n.a.  0.015  n.a.  n.a.  n.a.  n.a. | -0.354  n.a.  n.a.  n.a.  -0.223  n.a.  n.a.  n.a.  n.a. | 0.046  n.a.  n.a.  n.a.  0.214  n.a.  n.a.  n.a.  n.a. | 0.138  n.a.  n.a.  n.a.  0.894  n.a.  n.a.  n.a.  n.a. | -0.230  -0.302  -0.267  -0.119  -0.026  -0.243  0.075  -0.313  -0.124 | -0.426  -0.478  -0.419  -0.36  -0.262  -0.435  -0.207  -0.482  -0.388 | -0.018  -0.123  -0.089  0.126  0.183  -0.028  0.305  -0.154  0.142 | **0.034**  **0.005**  **0.013**  0.278  0.816  **0.025**  0.497  **0.004**  0.259 | -0.264  -0.325  -0.298  -0.134  -0.067  -0.272  0.041  -0.331  -0.149 | -0.459  -0.506  -0.458  -0.379  -0.307  -0.467  -0.231  -0.509  -0.407 | -0.047  -0.143  -0.107  0.106  0.148  -0.061  0.264  -0.162  0.115 | **0.015**  **0.002***  **0.006**  0.221  0.540  **0.012**  0.707  **0.002***  0.174 | -0.280  -0.316  -0.300  -0.113  -0.102  -0.266  n.a.  -0.315  -0.141 | -0.467  -0.492  -0.462  -0.361  -0.340  -0.456  n.a.  -0.500  -0.382 | -0.059  -0.135  -0.111  0.131  0.115  -0.049  n.a.  -0.132  0.117 | **0.010**  **0.003***  **0.005***  0.305  0.354  **0.014**  n.a.  **0.003***  0.197 | -0.286  n.a.  -0.269  n.a.  n.a.  -0.233  n.a.  n.a.  -0.111 | -0.476  n.a.  -0.437  n.a.  n.a.  -0.425  n.a.  n.a.  -0.338 | -0.061  n.a.  -0.076  n.a.  n.a.  -0.023  n.a.  n.a.  0.120 | **0.008**  n.a.  **0.013**  n.a.  n.a.  **0.032**  n.a.  n.a.  0.313 |

All results of partial correlation analyses between GWPC and GA, BW, and duration of ventilation for GWPC in VP/VLBW<FT with correlation coefficients, 95% confidence intervals, and p-values. Bold letters indicate statistical significance defined as p<0.05. Asterisks (*) indicate statistical significance defined as p<0.05, FDR-corrected.

Abbreviations: Bankssts, Banks of the superior temporal sulcus; BW, birth weight; GA, gestational age; inferiorparietal, Inferior parietal cortex; medialorbitofrontal, Medial orbitofrontal cortex; middletemporal, Middle temporal gyrus; n.a., not applicable; parsorbitalis, Inferior frontal gyrus, pars orbitalis; ROI, region of interest; superiortemporal, Superior temporal gyrus; supramarginal, Supramarginal gyrus.

**Table S7: Relationship between GWPC and birth variables for GWPC in VP/VLBW>FT**

| **Birth variable** | **ROI** | **10% projection fraction** | | | | **20% projection fraction** | | | | **30% projection fraction** | | | | **40% projection fraction** | | | | **50% projection fraction** | | | |
| --- | --- | --- | --- | --- | --- | --- | --- | --- | --- | --- | --- | --- | --- | --- | --- | --- | --- | --- | --- | --- | --- |
|  |  | **r** | **95% CI** | | **p-value** | **r** | **95% CI** | | **p-value** | **r** | **95% CI** | | **p-value** | **r** | **95% CI** | | **p-value** | **r** | **95% CI** | | **p-value** |
| GA | **Right hemisphere**  paracentral | -0.008 | -0.185 | 0.161 | 0.941 | -0.019 | -0.198 | 0.164 | 0.866 | -0.033 | -0.220 | 0.156 | 0.768 | -0.049 | -0.235 | 0.141 | 0.659 | -0.063 | -0.247 | 0.135 | 0.568 |
| BW | **Right hemisphere**  paracentral | 0.035 | -0.156 | 0.230 | 0.749 | 0.003 | -0.192 | 0.201 | 0.975 | -0.022 | -0.222 | 0.175 | 0.841 | -0.042 | -0.237 | 0.151 | 0.703 | -0.052 | -0.251 | 0.151 | 0.636 |
| Ventilation | **Right hemisphere**  paracentral | 0.050 | -0.181 | 0.267 | .  0.652 | 0.047 | -0.185 | 0.265 | 0.669 | 0.057 | -0.190 | 0.265 | 0.605 | 0.077 | -0.180 | 0.302 | 0.482 | 0.103 | -0.168 | 0.345 | 0.348 |

Correlation coefficients, 95% confidence intervals, and p-values of partial correlation analyses between GWPC in right paracentral lobule and GA, BW, and duration of ventilation. The results were not statistically significant (p<0.05).

Abbreviations: BW, birth weight; GA, gestational age; paracentral, Paracentral lobule; ROI, region of interest.

**Table S8: Relationship between GWPC and cognitive performance for GWPC in VP/VLBW<FT**

| **ROI** | **Cognitive performance** | **10% projection fraction** | | | | **20% projection fraction** | | | | **30% projection fraction** | | | | **40% projection fraction** | | | | **50% projection fraction** | | | |
| --- | --- | --- | --- | --- | --- | --- | --- | --- | --- | --- | --- | --- | --- | --- | --- | --- | --- | --- | --- | --- | --- |
|  |  | **r** | **95% CI** | | **p-value** | **r** | **95% CI** | | **p-value** | **r** | **95% CI** | | **p-value** | **r** | **95% CI** | | **p-value** | **r** | **95% CI** | | **p-value** |
| **Left hemisphere**  parsorbitalis  superiortemporal  **Right hemisphere**  bankssts  inferiorparietal  medialorbitofrontal  middletemporal  parsorbitalis  superiortemporal  supramarginal | Full-scale IQ | 0.118  n.a.  n.a.  n.a.  -0.028  n.a.  n.a.  n.a.  n.a. | -0.108  n.a.  n.a.  n.a.  -0.212  n.a.  n.a.  n.a.  n.a. | 0.353  n.a.  n.a.  n.a.  0.178  n.a.  n.a.  n.a.  n.a. | 0.291  n.a.  n.a.  n.a.  0.806  n.a.  n.a.  n.a.  n.a. | 0.140  0.019  -0.081  0.039  -0.043  0.097  -0.132  0.000  -0.003 | -0.102  -0.178  -0.276  -0.184  -0.228  -0.106  -0.350  -0.218  -0.240 | 0.386  0.237  0.116  0.282  0.165  0.314  0.107  0.225  0.243 | 0.210  0.863  0.471  0.726  0.699  0.384  0.237  0.997  0.980 | 0.136  0.034  -0.069  0.036  -0.042  0.104  -0.120  0.000  -0.007 | -0.103  -0.170  -0.261  -0.182  -0.236  -0.100  -0.350  -0.224  -0.235 | 0.376  0.255  0.130  0.271  0.166  0.313  0.109  0.228  0.239 | 0.222  0.763  0.541  0.749  0.711  0.351  0.283  0.997  0.948 | 0.123  0.032  -0.068  0.011  -0.032  0.086  n.a.  -0.023  -0.033 | -0.101  -0.167  -0.254  -0.199  -0.224  -0.117  n.a.  -0.241  -0.248 | 0.352  0.243  0.128  0.237  0.161  0.293  n.a.  0.207  0.202 | 0.270  0.776  0.546  0.921  0.774  0.441  n.a.  0.838  0.766 | 0.106  n.a.  -0.080  n.a.  n.a.  0.053  n.a.  n.a.  -0.074 | -0.118  n.a.  -0.262  n.a.  n.a.  -0.136  n.a.  n.a.  -0.274 | 0.330  n.a.  0.112  n.a.  n.a.  0.256  n.a.  n.a.  0.150 | 0.240  n.a.  0.338  n.a.  n.a.  0.097  n.a.  n.a.  0.865 |

All results of partial correlation analyses between GWPC and full-scale IQ for GWPC in VP/VLBW<FT with correlation coefficients, 95% confidence intervals, and p-values. The results were not statistically significant (p<0.05).

Abbreviations: Bankssts, Banks of the superior temporal sulcus; inferiorparietal, Inferior parietal cortex; IQ, intelligence quotient; medialorbitofrontal, Medial orbitofrontal cortex; middletemporal, Middle temporal gyrus; n.a., not applicable; parsorbitalis, Inferior frontal gyrus, pars orbitalis; ROI, region of interest; superiortemporal, Superior temporal gyrus; supramarginal, Supramarginal gyrus.

**Table S9: Relationship between GWPC and cognitive performance for GWPC in VP/VLBW>FT**

| **ROI** | **Cognitive performance** | **10% projection fraction** | | | | **20% projection fraction** | | | | **30% projection fraction** | | | | **40% projection fraction** | | | | **50% projection fraction** | | | |
| --- | --- | --- | --- | --- | --- | --- | --- | --- | --- | --- | --- | --- | --- | --- | --- | --- | --- | --- | --- | --- | --- |
|  |  | **r** | **95% CI** | | **p-value** | **r** | **95% CI** | | **p-value** | **r** | **95% CI** | | **p-value** | **r** | **95% CI** | | **p-value** | **r** | **95% CI** | | **p-value** |
| **Right hemisphere**  paracentral | Full-scale IQ | -0.219 | -0.445 | 0.009 | **0.048** | -0.221 | -0.434 | 0.015 | **0.046** | -0.228 | -0.447 | 0.004 | **0.039** | -0.239 | -0.451 | -0.004 | **0.031** | -0.250 | -0.463 | -0.013 | 0.568 |

All results of partial correlation analyses between GWPC in the right paracentral lobule and full-scale IQ with correlation coefficients, 95% confidence intervals, and p-values. Bold letters indicate statistical significance defined as p<0.05. * indicates statistical significance defined as p<0.05, FDR-corrected.

Abbreviations: IQ, intelligence quotient; n.a., not applicable; paracentral, Paracentral lobule; ROI, region of interest.

**Table S10: Comparison between VP/VLBW subjects with MRI data and without MRI data**

|  | **VP/VLBW with MRI (n=101)** | | **VP/VLBW without MRI (n=159)** | |  |
| --- | --- | --- | --- | --- | --- |
|  | **Mean** | **SD** | **Mean** | **SD** | **p-value** |
| **GA (weeks)** | 30.5 | ± 2.1 | 30.6 | ± 2.3 | 0.656 |
| **BW (g)** | 1324 | ± 313 | 1323 | ± 320 | 0.980 |
| **Full-scale IQ (a.u.) †** | 94.1 | ± 12.7 | 79.5 | ± 22.9 | **<0.001*** |

Statistical comparisons: GA, BW, and FS-IQ with two sample t-tests. Bold letters indicate statistical significance defined as p<0.05. Asterisks (*) indicate statistical significance defined as p<0.05, FDR-corrected.

Abbreviations: BW, birth weight; GA, gestational age; IQ, intelligence quotient; SD, standard deviation; MRI, magnetic resonance imaging; VP/VLBW, very preterm and/or very low birth weight.

† Data are based on 97 VP/VLBW subjects with MRI data and 120 VP/VLBW subjects without MRI data

**Supplemental References**

1. Eryigit Madzwamuse S, Baumann N, Jaekel J, Bartmann P, Wolke D. Neuro-cognitive performance of very preterm or very low birth weight adults at 26 years. *J Child Psychol Psychiatry*. 2015;56(8):857-864. doi:10.1111/jcpp.12358

2. Reyes LM, Jaekel J, Bartmann P, Wolke D. Peer Relationship Trajectories in Very Preterm and Term Individuals from Childhood to Early Adulthood. *Journal of Developmental & Behavioral Pediatrics*. 2021;Epub ahead. doi:10.1097/DBP.0000000000000949

3. Breeman LD, Jaekel J, Baumann N, Bartmann P, Wolke D. Preterm Cognitive Function Into Adulthood. *Pediatrics*. 2015;136(3):415-423. doi:10.1542/peds.2015-0608
